# Qualitative study for betel quid cessation among oral cancer patients

**DOI:** 10.1371/journal.pone.0199503

**Published:** 2018-07-17

**Authors:** Chen-Yi Lee, Chih-Feng Wu, Chun-Ming Chen, Yong-Yuan Chang

**Affiliations:** 1 Department of Oral Hygiene, College of Dental Medicine, Kaohsiung Medical University, Kaohsiung City, Taiwan; 2 Department of Medical Research, Kaohsiung Medical University Hospital, Kaohsiung City, Taiwan; 3 Division of Surgical Oncology, Department of Surgery, Kaohsiung Medical University Hospital, Kaohsiung City, Taiwan; 4 School of Medicine, College of Medicine, Kaohsiung Medical University, Kaohsiung City, Taiwan; 5 Division of Oral and Maxillofacial Surgery, Department of Dentistry, Kaohsiung Medical University Hospital, Kaohsiung City, Taiwan; 6 School of Dentistry, College of Dental Medicine, Kaohsiung Medical University, Kaohsiung City, Taiwan; 7 Department of Healthcare Administration and Medical Informatics, College of Health Sciences, Kaohsiung Medical University, Kaohsiung City, Taiwan; University of North Carolina at Chapel Hill, UNITED STATES

## Abstract

The psychoactive effects of using areca nut and its potential for dependence have been observed. However, the factors that create barriers to or promote chewing cessation are not well understood. This study aims to explore the behavioral changes of betel quid chewers who have been diagnosed with oral cancer within a transtheoretical model framework. Thirty oral cancer patients with betel quid chewing history were chosen for in-depth interviews. Qualitative content analysis was used to analyze the data and identify themes that described the behavioral changes of betel quid cessation. Our research showed that betel quid chewers with oral cancer typically experience four significant stages of behavior: pre-contemplation, contemplation, action, and maintenance. Each stage change was marked by specific characteristics. At first, chewers showed positive attitudes toward the psychoactive or social effects of betel quid. They then realized the negative effects of betel quid, such as dental or other physical problems. Some also realized that they were addicted to betel quid. When they decided to quit, most chewers reported going “cold turkey.” Some chewers successfully quit betel quid and attributed it to willpower. Those quitting because of the loss of oral functions were unable to chew anymore, though some chewers had experienced a relapse. In the maintenance stage, ex-chewers reported overcoming their addiction; however, relapse was possible. In this study, those who quit betel quid because of oral cancer usually quit tobacco and alcohol as well, with a lesser chance of recurrence. As the maintenance of chewing betel quid is multifactorial, this study provides information for betel quid cessation and oral cancer prevention.

## 1. Introduction

Areca nut is a common substance in Southeast Asia and the Asia Pacific region that is also used in migrant communities originating from these regions [1,2]. The psychoactive effects of areca nut use and its potential for dependence have been reviewed in previous literature [3,4]. Betel quid is a common preparation made from areca nut. The custom of chewing betel quid has existed for thousands of years since antiquity in Taiwan and Southern China. Moreover, areca nut has been widely used as a medicinal substance to treat diseases or ward off pestilence in traditional Chinese culture [5]. However, modern studies have shown that there is a strong relationship between areca nut/betel quid chewing and oral cancer [6]. Today, the chewing of betel quid is still prevalent among male Taiwanese and some aboriginal cultures under the influence of their social and cultural contexts [7–10].

Since the Cancer Control Act was promulgated and implemented in 2003, the Taiwan government has actively promoted cancer prevention. Prevention of oral cancer has been regarded as a major policy priority given its high prevalence due to betel quid chewing. Over the years, prevention has included oral mucosal screening in communities or hospitals, health education in communities or schools, health communication through the media, and a cessation program provided by public health nurses or hospital nurses [11,12]. However, the factors that create barriers to or promote chewing cessation are not well understood.

The transtheoretical model (TTM) is a theory applicable to the development of betel quid chewing cessation programs, as it is commonly used to explore addictive behaviors [13]. This study aims to explore within a TTM framework the behavioral changes of betel quid chewers who have been diagnosed with oral cancer. The main research questions are: (1) Can TTM be applied to describe changes in the behavior of oral cancer patients during the course of betel quid use? (2) What are the characteristics of oral cancer patients’ betel quid use behavior at various stages in the course of change? (3) Is an oral lesion process associated with betel quid addiction found in the qualitative data?

## 2. Materials and methods

### 2.1 Recruitment and sampling

Purposive sampling was adopted in this study and 30 participants were included. The criteria for inclusion of participants were oral cancer patients who have a history of habitual betel quid chewing, and who were capable of conducting a coherent conversation. The participants were recruited from the Cancer Center of Kaohsiung Medical University Hospital, which is located in southern Taiwan. A list of potential participants was provided by the attending doctors, and eligible patients were invited to participate in this study with informed consent. Participants received a gift card worth NT$500 as incentive for participation. The research protocol for this study was approved by the Human Experiment and Ethics Committee of the Chung-Ho Memorial Hospital, Kaohsiung Medical University.

### 2.2 Interviews and measures

The data were collected through individual semi-structured in-depth interviews. After providing informed consent, participants completed the Betel Quid Dependence Scale (BQDS), which is used to estimate the severity of addiction in betel quid chewers [14,15]. The interviews were conducted by a well-trained interviewer (TYC) who is very familiar with Southern Hokkien, a dialect widely used in Taiwan, especially among laborers. To facilitate the comfort of the interviewees, the interviews were conducted in a separate room that ensured the privacy of the participants. Interviews lasted between 20 and 60 minutes and were digitally recorded, transcribed, and checked for accuracy. All participants agreed to audio and video recording.

The interview guide included the following concepts (as shown in Table 1), in order: (1) initiation of the use of betel quid; (2) the situations that facilitated continued chewing after first trying betel quid; (3) the changes in betel quid usage; (4) the effects of long-term chewing; (5) previous experiences with quitting betel quid; (6) the circumstances around quitting betel quid and the maintenance of cessation; (7) situations in which relapse occurred.

**Table 1:** The interview guide.

Concepts	Examples of interview question	Description
(1) Initiation of the use of betel quid	“Can you talk about the situation in which you chewed betel quid for the first time?” “Why did you want to try it?	
(2) the situations that facilitated continued chewing after first trying betel quid	“What made you continue chewing after first trying it?” “How did it feel?”	
(3) the changes in betel quid usage	“Do you have any preference for the type of betel quid you use?” “Did you increase the amount of usage gradually?”	
(4) the effects of long-term chewing	“What are the effects long-term chewing has on you?”	
(5) previous experiences with quitting betel quid	“Have you quit betel quid before?” “How many times did you quit?” “Why did you want to quit?” “How did you quit?”	In this study, all participants have experiences quitting betel quid.
(6) the circumstances around quitting betel quid and the maintenance of cessation	“How long was your longest period of cessation?” “Do you feel strange when stopping chewing betel quid? Do you suffer from sleepiness or anxiety?” “What are the causes of successful cessation for you?” “Do you stop chewing completely?”	For those participants who have experience successfully quitting betel quid.
(7) the situations in which relapse occurred	“After you quit betel quid, what is the reason you started chewing again?”	For those who have experienced relapse.

Note: The interviewees are oral cancer patients, so the interviewer must clarify whether the interviewees’ time node of betel quid cessation was before or after the time when they got cancer, and whether they were forced to quit betel quid due to insufficient oral functions.

### 2.3 Analysis

**Fig 1:**
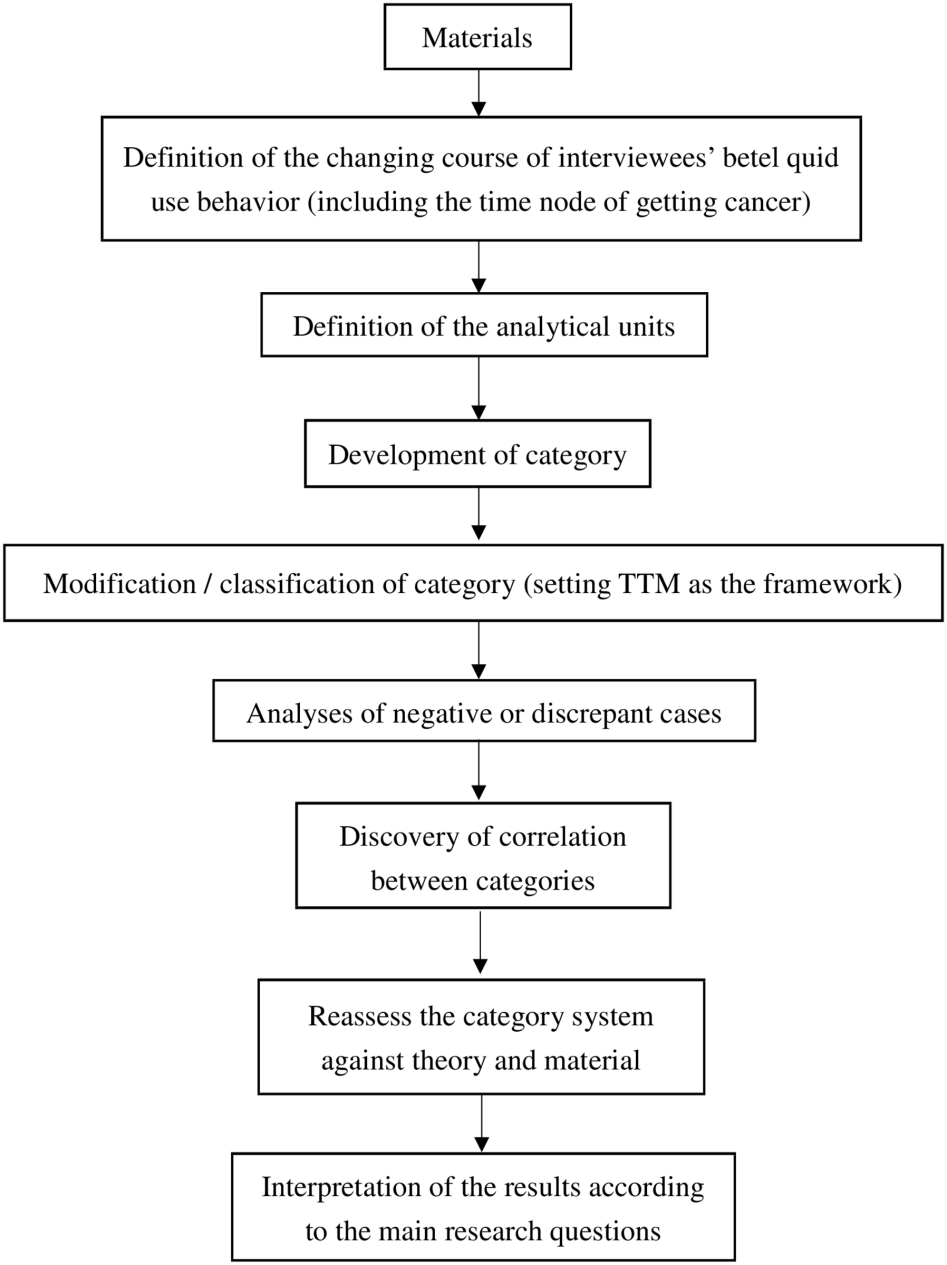
Flow diagram for the steps of the analysis of the qualitative data.

Qualitative content analysis was conducted using a constant comparative method to categorize the description of participants into five stages of change according to the TTM framework. We then modified the model according to the phenomena we observed among the participants. The steps of the analysis of the qualitative data are presented in Fig 1.

All transcripts were analyzed by the first author (CYL). A month after the initial analysis was finished, the researcher randomly selected 10 transcripts to reanalyze and found consistent results. Another researcher (YYC) separately randomly selected 6 transcripts to ensure the consistency of the categories and definitions of data analysis. The attending doctors (CFW and CMC) were invited to investigate the appropriateness of the text interpretation.

## 3. Results

All 30 participants were non-aboriginal males. Among them, 20 patients suffered from oral cancer in the buccal mucosa (66.7%), 5 suffered from tongue cancer (16.7%), and 5 suffered from other forms of oral cancer (16.7%). The mean age was 53 years (ranging from 35 to 72 years old). The history of chewing betel quid ranged from 15 to 50 years. The BQDS score of most interviewees (90.0%) was higher than 4, and they were suspected of reaching the diagnostic criteria of betel quid addiction; the score of 17 interviewees (56.7%) was higher than 8, and they were suspected to be severe betel quid addicts. Of the 30 participants, 26 of them have quit betel quid and achieved the maintenance stage before the study period, while only 4 participants remained in the action stage during the study period. The duration of time since quitting ranged from 0.17 to 26 years. The participants can roughly be divided into two types: (1) chewers who started from the pre-contemplation or contemplation stage, moved straight to the action stage due to oral cancer, and then possibly achieved the maintenance stage; and (2) chewers who had tried to quit betel quid but possibly experienced a cycle of chewing and quitting, then possibly achieved the maintenance stage before or after they were diagnosed with oral cancer (Table 2).

**Table 2:** The characteristics of participants.

Case No.	Age	Occupation	Cancer site	BQDS score	Type	No. of times trying to quit	Stages of change, and timing of diagnosis of oral cancer	Main reasons for quitting	Time since quitting
M1	60	Car or excavator driver	Lower gum	12	1	1	PC→[ca]→A→M	Oral cancer	10 years
M2	35	Laborer	Buccal	15	2	2	PC→C→A→M→[r]→PC→[ca]→A→M	Prevent children from following my example; oral cancer	2 years
M3	36	Hydropower project	Tongue	13	2	3	PC→C→A→[r]×2→PC→C→A→[ca]	*Vibrio vulnificus*; strange feeling in the mouth, repeated mouth injury; tongue cancer	2 months
M4	55	Formwork	Buccal	4	2	3	PC→C→A→[r]→PC→C→A→[r]→PC→C→[ca]→[l]→A→M	A mouth injury often hurts; oral cancer	1 year and 4 months
M5	72	Heavy truck driver	Buccal	6	2	Several dozens	PC→C→A→[r]sd→C→A→M→[ca]	Others laugh at my black mouth; made a vow to quit if Frank Hsieh was elected in 2003	10 years
M6	53	Cutting and welding steel house, driver	Buccal	7	2	1	PC→C(p)→A→M→[ca]	The mouth can’t be opened (OSF)	10 years
M7	66	Laborer	Buccal	10	1	1	PC→[ca]→[l]→A→M	Oral cancer	More than 2 years
M8	69	Heavy truck driver	Buccal	10	1	1	PC→C→[ca]→A→M	Oral cancer	4 years
M9	38	Factory worker, truck driver	Tongue	7	2	5	PC→C→A→[r]×4→C→[ca]→A→M	Stopped eating when the mouth was broken; tongue cancer	More than 1 year
M10	64	Farming (landowner), rarely goes out to work (rich family), member of temple committee	Buccal	9	2	5–10	PC→C→A→[r]<10→C→[ca] →[l]→A→M	It doesn’t taste good; hit the jackpot (oral cancer); all teeth were lost	2 years
M11	46	Driving a heavy truck at night	Lower gum	7	1	1	PC→[ca]→A→M	Oral cancer and surgery	4.5 years
M12	60	Seafood business	Buccal	13	2	Several dozen	PC→C→A→[r]sd→stroke→A→M→[ca]	Others told me to quit; stroke	3 to 4 years
M13	64	Business, truck driving around Taiwan	Buccal	10	2	2	PC→C→A→[r]→C→[ca]→A→M	My wife blamed me; oral cancer; my daughter blamed me	11 months
M14	60	Business in steel structures	Buccal	7	2	1	PC→C→[l]→A→M→[ca]	Toothache; no strength	5 to 6 years
M15	51	Rarely goes to work (rich family)	Retromolar	8	2	1	PC→C→(p)→[l]→A→M→[ca]	Toothache, unable to chew; the mouth can’t open (OSF)	More than 7 years
M16	51	Truck driver	Tongue	8	2	1	PC→C(p)→A→M→[ca]	Leukoplakia; oral cancer	6 to 7 years
M17	56	Different occupations, driving truck long distances over long periods	Buccal	1	2	2	PC→C→A→M→[r]→M→[ca]	Fatty liver index; others look at me differently	26 years
M18	56	Driving truck long distances	Mouth floor	12	2	2	PC→C→A→[r]→ PC→C→A→M→[ca]	It’s too expensive; the mouth abscesses cannot heal	5 years
M19	60	Master of metal craft	Buccal	13	2	4	PC→C→A→[r]×3→C→A→M→[ca]	Suffer from indigestion, diarrhea, mouth injury, uncleanliness, bad appearance, tooth abrasion, and powerless lower jaw	8 years
M20	50	Delivering goods with motorcycle, business	Buccal	5	2	3	PC→(r)→A→M→[r]→PC→(r)→A→M→[r]→PC→[ca]→A	Unable to buy it when going abroad (mainland, Japan); oral cancer	4 months
M21	51	Mechanical processing	Buccal	3	2	1	PC→C→A→M→[ca]	It costs a lot; my life changes, and I don’t need betel quid to refresh myself	13 to 14 years
M22	44	Business in cement and air conditioning	Buccal	6	1	1	PC→[ca]→A→M	Oral cancer	4 to 5 years
M23	66	Delivering goods via truck	Lower gum	11	1	1	PC→[ca]→[l]→A→M→[ca]	Esophageal cancer	13 years
M24	41	Hydropower project, soldier, workman	Buccal	4	2	2	PC→C→A→M→[r]→C→[l]→A→M→[ca]	Broken mouth and toothache	7 years
M25	49	Works on a construction site	Buccal	3	2	At least 10	PC→C→A→[r]×10→C→A→M→[ca]	I can’t make a good impression on others when communicating with them at work	8 years
M26	54	Plastic processing	Buccal	15	1	1	PC→C→PC→[ca]→A→M	My wife was opposed; oral cancer	Less than 1 year
M27	55	Painter	Buccal	5	2	1	PC→[l]→A→M→[ca]	My teeth fell out due to chewing betel quid	16 years
M28	40	Excavator driver	Tongue	15	2	At least 20	PC→C→A→[r]>20→[ca]→A	Bad appearance of mouth; tongue cancer	Almost a year
M29	52	Concrete truck driver	Tongue	12	2	Several dozen	PC→C→A→[r]sd→[ca]→[l]→A→M	It costs a lot; go to the toilet frequently; unable to eat due to the pain; oral cancer	5 years
M30	52	Handyman	Buccal	11	2	2	PC→C→A→[r]→C→[ca]→A	It hurts the teeth and gingiva; oral lesions; diarrhea	7 months

Type: 1 = chewers who started from the pre-contemplation or contemplation stage, moved straight to the action stage due to oral cancer, and then possibly achieved the maintenance stage; 2 = chewers who have tried to quit betel quid, but possibly experienced a cycle of chewing and quitting, then possibly achieved the maintenance stage before or after they were diagnosed with oral cancer; (p) = precancer; (r) = restricted environment; PC = pre-contemplation; C = contemplation; P = preparation; A = action; M = maintenance; [ca] = cancer; [r] = relapse or recurrence; sd = several dozen; [l] = loss of oral functions.

**Fig 2:**
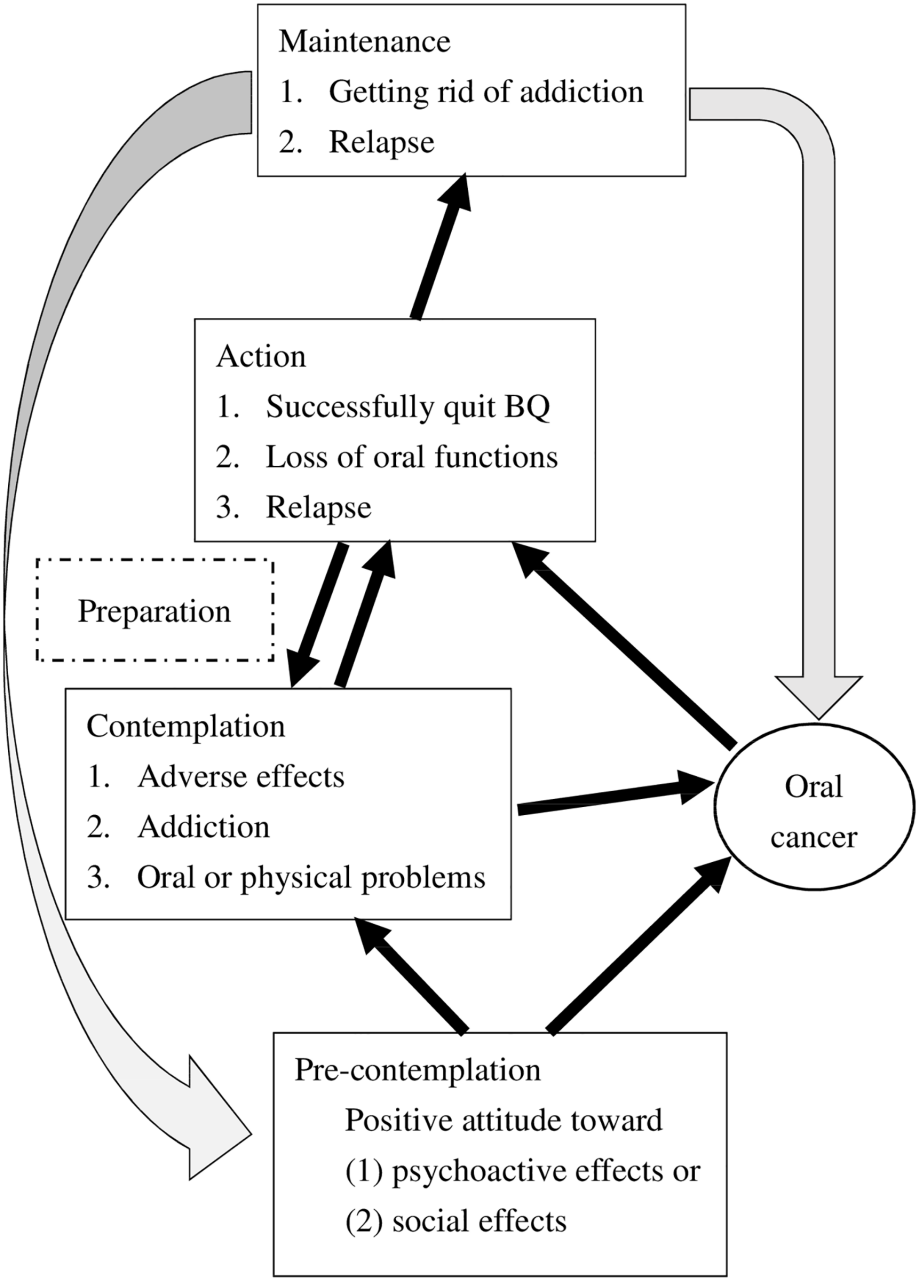
Processes in the stages of change in the transtheoretical model (TTM). The curved arrow represents recurrence, and the right-angled arrow represents the diagnosis of cancer some time after quitting BQ.

The participants typically experienced four stages of behavior: pre-contemplation, contemplation, action, and maintenance. Each stage change was marked by specific characteristics. However, the characteristics of the preparation stage were insignificant among this sample (as shown in Fig 2). Quotes indicating interviewees’ characteristics at various stages are provided in the S1 Appendix.

### 3.1 Pre-contemplation stage

The participants lacked motivation for change. They had a positive attitude toward chewing betel quid, and most of them mentioned its psychostimulant effect of “being refreshing.” In particular, chewing betel quid was very helpful in keeping them awake when driving long distances. In addition, chewing betel quid also helped relieve them of boredom or stress and kept them warm in cold weather. Furthermore, chewing betel quid is a social mechanism, and the use of betel quid was sometimes unavoidable in social interactions.

#### 3.1.1 Positive attitude toward psychoactive effects

The participants believed that betel quid produces positive effects such as keeping them awake, relieving boredom, suppressing their thirst, preventing the body from getting cold, and relieving stress. For example, some participant descriptions are listed below.

M1: “The feeling of eating betel quid is that of chewing continuously. When there is something to chew, you are less likely to get bored, you also feel less thirsty, and your mouth is less dry. Chewing then continues.” M1 also said, “When driving, chewing will reduce boredom and sleepiness and refresh the mind.”

M14: “Oh … Betel quid chewing helps the body to stay warm and tolerate cold weather.”

M11: “I feel betel quid [use] is because of my job as a driver …. My emotions … I am more …. My personality is more rushed, impulsive, and more emotional. I sometimes lose my temper easily. When I lose my temper, I will naturally want to smoke tobacco with betel quid to calm my emotion.”

Some interviewees expressed that betel quid chewing was a habit. For instance, M10 said, “There is nothing to speak of. It does not taste good or bad. That’s it and it is just a habit.”

It is a hobby for some interviewees. For example, M7 said, “The more you chew, the more you like it. Sometimes, I even sleep with betel quid in my mouth [laughs].” Some interviewees especially like the feeling of chewing betel quid and smoking together. M23 said, “I have never thought about it …. Just smoking tobacco along with chewing. Chewing betel quids while smoking is more interesting, so I do not want to make any changes … If I did not have this disease [oral cancer], this would not have changed.”

#### 3.1.2 Positive attitude toward social effects

The participants believed that chewing betel quid has a social nature and is part of life. In response to the question, “Do you want to quit?” M22 said, “Ah no … because I have changed my job to air-conditioning, haven’t I? In air-conditioning, my seniors chew more betel quid than I do. When both of us have a day out, we cannot do without betel quid … If I go to your home today to install air-conditioning, and you give me some betel quid, tell me how I can change this? It is also a kind of … how should I say this? When you visit a client’s house and they are chewing betel quid … this is considered socializing ….”

M24 also said, “Because when you are at work, you will definitely buy some for your masters. The so-called master is the person who teaches you. You buy some betel quid, and the master buys some; you need to buy some too. This counts as showing him respect, so you should treat him.” M24 considered, “This is the culture of Taiwan, and we have to do three things after getting off work [cigarettes, wine and betel quid] … For instance, at the construction site, they often buy beer during work, and chew betel quid, smoke cigarettes, and drink wine at the same time…”

### 3.2 Contemplation stage

Generally speaking, the participants believed that chewing betel quid would not impair their interpersonal relationships or family and social functioning; however, it has adverse effects on their appearance, dental function, and oral mucosa. Furthermore, the participants also believed that betel quid is addictive and causes a financial burden when the usage is high. Physical problems were also a reason for quitting betel quid.

#### 3.2.1 The adverse effects of chewing betel quid

Chewing betel quid often damaged the chewer’s oral mucosa and led to oral ulceration. The participants explained that the oral ulceration caused them strong pain when they were eating, so they usually stopped chewing for several days and began chewing again when the mucosa recovered. For example, participant M4 said, “Ah, chewing betel quid sometimes makes holes in my mouth. It is painful. If it is painful, then I quit. If I quit chewing and recover, and see others chewing betel quid, I will go buy some again. Just like this.”

M9 described, “I came across this in my social life … I also think … it is annoying. You asked if I wanted to quit; yes, sometimes. I stop chewing if there are holes in my mouth. When it recovers, I start chewing again.”

Long-term chewing of betel quid has also led to tooth abrasion, insufficient chewing force, and gingiva swelling. The combination of areca nut and slaked lime also produces a reddish discoloration of the mouth and saliva; some participants were troubled by their appearance.

Due to long-term chewing of betel quid, M19 has experienced weakness in the lower jaw and difficulty in chewing meat. This has bothered him: “Not hygienic! My teeth became ugly and very black, and all of them fell out. The molars were not working, falling out, and were weak … Betel quid chewers have a weak lower jaw. It is difficult to chew a piece of meat even when you want to. I can chew betel quid no matter how hard it is, but I cannot chew meat ….”

M25 believed chewing betel quid makes a poor impression on others, which led him to think of quitting betel quid: “When meeting others for work, the impression is not very nice. We are considered the middle level at the construction site, but there are more senior levels. When talking to people, or whatever it is, the impression is not very good.”

Interviewees often mentioned another bad effect, which is diarrhea. M29 said, “It makes people suffer from diarrhea…Everybody is like this, and often goes to the toilet. The more you eat, the more frequently you go to the toilet.” Most interviewees who habitually drank alcohol stated that it was easy to swallow the betel quid when you drink at the same time. Therefore, they often smoke when chewing betel quid, and drink after spitting out the betel quid. M22 said, “Ah, maybe my stomach is not good. Ah, sometimes, you will swallow the betel quid residues, just like the balloons. Sometimes, you will swallow it if you are careless…Residues will be swallowed more or less, and it is bad for your stomach…”

Additionally, the production of betel quid is seasonal. If there is high yield, the price will be cheaper and more betel quid will be packed in one bag for 50 TWD. When there is lower yield, there are fewer betel quid packed in a bag for the same price. For the interviewees with high usage, they considered the expense of buying betel quid a financial burden. M18 said, “Sometimes when I think, ‘Ah, it is becoming more expensive,’ I want to quit. Spending a few hundred a day.”

Most interviewees have most of the above problems, but some interviewees do not wish to quit betel quid. For example, M20, M22 and M23 intend never to quit betel quid. M10 quit because he thought that it was not tasty, but he never felt any bad influence.

#### 3.2.2 Addiction

The participants generally believed that betel quids are addictive. Just like smoking, chewing is a “habit” to them. Addicted chewers hesitated to quit and were controlled by their craving for betel quid.

M30 described it this way, “Sometimes I want to, but cannot quit. Anyway, many people, like your mother, have been telling you not to chew betel quid, but you still cannot quit. Since betel quid chewing has already become my habit, I have to chew them.” M30 also said, “This … regarding betel quid chewing … it only has a hundred harms, and no benefit. Even if you wanted to, you cannot give it up, even if you wanted to stop chewing every day. You want to stop chewing tomorrow, but … but you will just continue chewing. If you think about not chewing tomorrow, before bed you think about not chewing any betel quid tomorrow. You will still … you will still chew some the next morning.”

M18 also said, “A habit, if it is a habit, you will not know what its pros and cons are anymore. I do not know, but it is a habit. I feel like having something to chew when driving at work, chewing one after another. I do not feel anything much, but I just like chewing. When my mouth is empty, I feel like finding something to chew.”

Even people who don’t like the taste of betel quid can get addicted to it after trying it several times. For instance, M12 encountered betel quid due to social engagement, “It smells bad, and is not tasty [laughs]. I felt like throwing up after chewing it, and learned to eat betel quid the first time… (Interview: What did you feel later?) It does not…does not smell so bad, but…almost the same…it’s always that taste after I tried ten or twenty times. It has a bad smell…but later I got addicted, and cannot quit.” Not a few interviewees indicated that they would chew betel quids one after another when becoming addicted. They often ate more over time, and some interviewees chewed betel quids all day and even slept with betel quid in their mouths. For example, M19 said, “I chew betel quid all day except during sleep, and chew every moment… Sometimes, I even sleep with betel quid in my mouth, because it has become a habit.” Most interviewees believed that it was a bad habit to sleep with betel quid in their mouths, but a few interviewees, such as M7 and M27, still never thought of quitting betel quid since they were seriously addicted.

#### 3.2.3 Physical problems

In this study, participants with oral cancer suffered a tremendous psychological impact, though they usually started quitting betel quid, cigarettes, and alcohol together immediately after receiving the cancer diagnosis. Therefore, we adopted “oral cancer” as an independent factor, as shown in Fig 2.

Other physical problems probably motivated chewers to quit betel quid. For example, M12 said, “I have had a stroke …I have been quitting for around three to four years….” M17 said, “I have to collect my medication from the Hepatology Department, and my fatty liver index is relatively high. Since it became relatively high, my doctor has told me not to chew it anymore and also to quit tobacco ….”

### 3.3 Preparation stage

The participants typically showed insignificant characteristics in the preparation stage; most of them reported taking action immediately when they decided to give up chewing betel quid. For example, M21 stated, “You won’t believe it, even if I say it. I was thinking when driving, I was thinking about how much I had spent on eating betel quid in these many years, and on smoking tobacco. Why I do eat betel quid? … I then threw them away and quit eating them from then on. If people force you, you will experience a rebound. You will follow it if it comes from your inner self ….”

### 3.4 Action stage

Once the participants made up their mind, most of them reported quitting betel quid by going “cold turkey” and practicing self-restraint, willpower, substitution, or reduction. Some of the participants quit chewing because they were restricted by oral lesions or insufficient chewing function. Some participants successfully quit betel quid, but some participants experienced relapses.

#### 3.4.1 Successfully quit betel quid

Many interviewees in this study were determined to quit betel quid after learning that they suffered from cancer or precancerous lesions, and their determination to quit betel quid was strengthened. For example, M26 said, “Actually, I’d already known this two months before the examination. I stopped eating it in March 2014, and I knew that my mouth could not be saved. I know this fairly well, since I had chewed so many betel quids. It must be oral cancer, so I have to face it by myself…”

The most common strategy was “going cold turkey.” A few participants quit by reducing the amount gradually and some of them used substitutes such as chewing gum, but they concluded that all kinds of substitute are useless; the most effective method is willpower.

M11 said, “Use willpower, just like smoking. Even now I still want to smoke tobacco. When I smell tobacco, I still have that urge to smoke. How amazing it would be to have just one smoke. However, I used my willpower to overcome my addiction to smoking. Similarly, for betel quid, I used willpower to quit and it worked. When I said no, it meant no.”

Some interviewees experienced withdrawal symptoms when trying to quit betel quid. M13 said, “I felt strange in my mouth if I did not eat betel quid, and wanted to chew betel quid all the time. Later, I tried to control myself, and got used to it in more than one month.”

M12 also said, “It needs determination. Some friends may deliberately make fun of you and give you some. I will say no, and it means no. Because… doing business until my stroke, I told myself not to chew any betel quid. (Interviewer: How about when people pass them to you?) When I said no, it means no. Because I would tell him I had a stroke.”

M19 said, “Oh, my method does not work on many other types, only this. Whenever you want to chew betel quid, quickly brush your teeth and make them feel clean. With a clean mouth, you are less likely to eat anything. Even if people ask me to eat peanuts and chew tea leaves, it will not work. I have tried quitting several times by those methods, which were not effective.”

M21 believed it is related to environmental changes: “It should be said the social life and work load have been reduced. When you were young, like at 20 and 30 years old, you might have had a large workload or needed to earn money; you needed to energize yourself. Turning 40, when turning almost 40 years old, your income and everything will stabilize and you not need to put in that much effort. Of course, if you do not have much physical output, you will not need to energize yourself to an excited state ….”

M17, M21, and M25 are interviewees whose BQDS score is lower than 4 points. M17 said, “…I’m not addicted.” M21 believed, “I’m a little bit addicted, but it is not severe…it is tiny, and my addiction is tiny. I want to chew betel quid, but the quantity … I will control the amount and I can refrain from it. I can survive without it.” It seems that as long as reasons for social use are overcome, the chances of quitting betel quid can be increased. However, M25 tried to quit at least 10 times. M25 considered that he could quit betel quid successfully because, “I had a stable job and stable customers at that time. I just needed to make an inspection tour at the construction site. If [the workers] need some, I will prepare some for them. I don’t eat anymore.” Hence, before the interviewees’ occupational environment changes, the reasons for use due to social contacts still have a significant influence.

Being unable to obtain betel quids is also considered a reason for quitting. M20 said, “It is because you cannot buy them overseas, which has happened twice. You need to do business overseas and cannot buy any, so you will quit it naturally when you return.” However, M20 did not intend to quit betel quid at that time and returned to chewing betel quid after coming back two times. M3, M20, M28, and M30 were still at the action stage. They either completely quit or decreased the amount, and still had not entered the maintenance stage.

#### 3.4.2 Loss of oral functions

Some participants were restricted by loss of oral functions and had to quit betel quid. For example, M14 said, “Just oral pain. The teeth were weak when chewing and I was unable to continue to chew, and that made me quit.” M24 also had a similar experience: “I started chewing on this side. When the teeth had fallen out and I could not chew, then I moved to the other side. When that side also started loosening up, and I was also in pain, I decided to stop chewing ….” M15 quit betel quid gradually due to oral submucous fibrosis: “In the end, I was … my mouth could not open, and that made it difficult to squeeze any betel quid in, you know, then I chewed less. I tried to squeeze them in, but had less than 10 a day. It hurt when chewing ….” M23 quit when hospitalized after being diagnosed with cancer: “Your mouth cannot chew after having esophageal cancer, the feeding tube will be inserted here for feeding; how can you chew betel quid?”

#### 3.4.3 Relapse

Some participants have tried to quit betel quid before they lose their oral functions, but had recurrent relapses. The common reasons for recurrence were “weak willpower,” “cravings,” and “social intercourse.” For example, M5 said, “Err … Sometimes several months, sometimes I relapsed after several weeks. It is just like taking medication. You think about it when you stop having it. The willpower helps to stop you from chewing … Ah … It is very boring every day. If for others … my friends were talking and chewing, and offering some betel quid. I was embarrassed to turn them down…”

Another participant, M28, tried to quit betel quid more than twenty times. He said, “Have tried to quit too many times. The craving comes almost three days to one week after quitting. I am working at this construction site where every colleague chews them. We treat each other, so I feel embarrassed if I do not take it. So I will take it and squeeze them into my mouth.”

M12 mentioned a rebound effect in his experience of quitting betel quid: “I have tried quitting chewing betel quid dozens of times. However, chewing increases every time after quitting. I ended up having more every time after I quit, and this cycle continues. If I quit now, I will still want to chew, and will even have a lot more than I did before … The more I quit, the more betel quid I will chew.” M12 finally succeeded when he had a stroke.

Furthermore, M30 mentioned the uselessness of substitutes: “There is no way to quit. This is me … I have no idea how to quit. If you asked me to try chewing gum, I would not get used to chewing gum, I would also not get used to chewing candies. Also, you can buy something … I forget … lico … lico … licorice, right! Buying licorice cannot help, I cannot get used to it. It is the same with sugar cane … Every day you think about not having any the next day, and that you will not have any tomorrow morning. However, you will still buy betel quid to chew as usual when you wake up. Many people have said not to chew betel quid, but I will not listen. They said their own things, and I continue my chewing.”

Some participants reported that long-term chewers often suffer discomfort in their teeth if they abruptly stop chewing; hence, they continued to relieve the pain in their teeth by chewing. M29 said, “Not able to quit. My mouth becomes dry. The tooth will become swollen, and that hurts. The pain is relieved when chewing ….” M29 had tried quitting dozens of times before he had oral cancer. He quit after all his teeth had been extracted.

### 3.5 Maintenance stage

The participants successfully got rid of the temptation to chew betel quid; they had no more craving for chewing and no more withdrawal symptoms. In this study, many participants quit betel quid due to oral cancer or poor oral functioning; most of them reported that they would no longer use it. Some participants who quit betel quid and achieved the maintenance stage through other motivations reported a higher chance of recurrence. In any case, those with oral cancer suffered a tremendous psychological impact, though they usually started quitting betel quid, cigarettes, and alcohol together immediately after receiving cancer diagnosis.

#### 3.5.1 No withdrawal symptoms and overcoming the addiction

The participants insisted on stopping chewing betel quid through willpower, whereby they passed through the difficult period of withdrawal symptoms and no longer suffered the craving to chew. For example, M13 said, “Coming here for chemotherapy … My daughter did not let me chew betel quid … After having a strange feeling for almost one to two months, this feeling went away after I got used to it … Quitting causes a strange and itchy feeling in the mouth; it creates a craving for chewing. Later, I kept tolerating and tolerating this until I got used to it after a month. I may feel [quite tired], and after approximately … more than a month, everything went back to normal.”

In response to a question about situations where the participants were with friends and their friends were chewing, M10 said, “… I say no, and that means no. If you want to, you can chew as much as you want. If you rush to have it, then just go! I have no way to persuade you guys.” M14 said, “In the past, I got used to chewing. I would find it and would have thoughts about it. Now, I will not think about it as I have stopped chewing; I have no intention to buy it or whatever.”

M11 attributed his ability to quit successfully to being scared by cancer, and was completely free of cravings: “Because I … honestly speaking, I was scared … This cancer is really … this … my daily routine had been completely changed. You dare not eat what you like. For myself, I was engaged in my social life, and everything had been changed. Ah … [sighs]. Therefore … betel quid chewing is really not good. Life and daily routine will have changed ….”

One of the interviewees said that he would still crave betel quid in his mouth but dared not eat it. M19 said, “I just, just want to have a taste. It is nothing, and all people can be greedy in their mouths. I would still dream of chewing betel quid two or three years later after quitting. I don’t know why I dreamed about it after quitting.” In addition, M24 and M25 did not show any withdrawal symptoms owing to their low addiction level. Generally speaking, the degree and duration of abstinence symptoms show individual differences.

#### 3.5.2 Recurrence

Some participants reported that they had reached the maintenance stage, and also had experienced a recurrence before they had been diagnosed with cancer. The influencing factors included “irritable mood” and “work site culture.”

M2 began chewing again to relieve stress: “I forgot [answering with a smile]. I was feeling annoyed, or whatever it was during that period of time … and started chewing again. Chewing and then … it continued … and continued chewing, and it did not stop.”

M24 began chewing again because of the culture at his work site. “Sometimes when I work at the work site, people treat you… Chewing again will result in addiction. Just like this ….”

It is common to use betel quids as a social mechanism in electioneering, especially among the aborigines who live in Southern Taiwan. M17 began chewing again because of the contact with aborigines during the election period: “… because I have a godbrother who is an aborigine. He is the chairman of the village representative council, so like him—he sometimes runs elections or something—I will visit each of their villages, for example ten villages in Sanmin Township. We will even visit mountain villages in Pingtung County. I will chew some during that time. If I do chew, but … that would be … just for a very short period of time.” M17 was not addicted before quitting betel quid and relapse was not caused when he chewed occasionally. But this case also shows that culture and social contact might make betel quid quitters chew again.

## 4. Discussion

The research findings showed that the changes in the addiction behaviors of betel quid chewers showed noteworthy characteristics in the pre-contemplation, contemplation, action, and maintenance stages; characteristics in the preparation stage were less important. Some of the interviewees were in the pre-contemplation stage (did or did not achieve the contemplation stage), moved straight into the action stage, and then into the maintenance stage due to their cancer diagnosis. Some of them quit chewing betel quid spontaneously and experienced a cycle of chewing and quitting during the contemplation and action stages. They later moved directly to the action stage and achieved the maintenance stage due to cancer. Some also quit betel quid spontaneously and had already achieved the maintenance stage several years before being diagnosed with cancer. This study found that relapse occurred easily between the contemplation and action stages. Even those who entered the maintenance stage could still revert to the pre-contemplation stage. This phenomenon, however, was rarely observed in betel quid quitters who only quit after being diagnosed with cancer. This indicates that the life changes brought about by the cancer diagnosis and follow-up treatments resulted in a significant psychological impact on the chewers, which reduced the likelihood of relapse after quitting.

This research found that chewers of betel quid in the pre-contemplation stage showed positive expectations and dependence on the effects of betel quid, which included “a refreshing feeling,” “killing boredom,” and “stress relief.” Moreover, there were positive expectations of the social function of betel quid, consistent with previous studies [16,17]. At this stage, they had not experienced any negative impacts associated with betel quid and thus lacked the motivation to change. Therefore, we recommend that health education include information regarding the negative impacts described by chewers in the contemplation stage and by cancer patients. This could increase the motivation of chewers in the pre-contemplation stage to make changes.

As for the chewers in the contemplation stage, they had begun to realize the negative impact caused by chewing betel quid. The most common consequence was pain caused by repeated “broken mouth.” Long-term chewing of betel quid also caused tooth wear, weakening of the lower jaw, and accelerated tooth loss, as well as poor appearance, blackened teeth, and other problems. In addition, the realization that they were addicted was considered a main characteristic. A strong craving for betel quid is also a common sign of addiction recognized by research in other countries, and habitual daily use is a universal phenomenon [1]. After they became addicted, the increased usage also meant that betel quid became a significant expense. Hence, the positive and negative impacts began to conflict. Repetitive oral mucosa damage could also lead to mucosal tissue mutation in the long term and increase the likelihood of developing cancer [18]. Thus, the government should address the potential food safety issue of its use [5].

This study found that the chewers of betel quid did not show significant characteristics in the preparation stage. No participant mentioned chewing cessation programs, suggesting that although there are effective betel quid cessation programs in Taiwan [12], they need to be promoted to increase their use. Most of them moved straight to the action stage when they decided to quit. However, the duration of the action stage varied. Some people could persist for three to four months, while others only persisted for several days. “Stop chewing completely” and “willpower” were the main methods adopted to quit betel quid. Chewing gum and other substitutes were found to be ineffective. Being able to reject the temptation offered by friends in social situations was also considered a main factor in quitting betel quid. By studying the descriptions of chewers with repeated relapses, it was found that addiction and social interactions led to diminished will power, which was the main factor for relapse in the action stage. Furthermore, during the process of quitting, a rebound effect in the amount of betel quid needed may develop alongside the increased frequency of efforts to quit. Hence, enhancing the self-efficacy of betel quid quitters is an important issue.

Some interviewees mentioned that after long-term betel quid chewing their teeth became swollen and painful when they stopped chewing suddenly. This stopped once they began chewing again. While the mechanism behind this phenomenon is still unknown, it might be related to oral mechanics and oral hygiene. Long-term chewing may affect the bones that support the teeth. Quitting chewing suddenly may reduce the usual forces generated by the teeth and jawbone, resulting in a rebound effect and discomfort. For patients with poor oral health and periodontal disease, the chemicals in betel quid may help relieve their discomfort through antibacterial activities [19,20]. After the patient stops chewing betel quid, gum inflammation continues, causing loose teeth. This could discourage chewers from quitting betel quid. However, this claim is still controversial [21,22]. In any case, this phenomenon may lead to the failure to quit betel quid, and thus the mechanism involved merits further exploration in future research. Therefore, inter-professional collaboration in betel quid cessation programs and the participation of dental professionals may improve the success rate of cessation.

Regardless of whether it was due to cancer or for other reasons, those who successfully quit betel quid were free from addiction and withdrawal symptoms after entering the maintenance stage. Furthermore, they were able to refuse the temptation offered by others and no longer craved betel quid. Chewers who quit due to cancer generally displayed strong determination and were less likely to experience signs of relapse. As for betel quid chewers who quit before being diagnosed with cancer, they were more likely to experience relapse during the maintenance stage. This was due to the expected positive effects of chewing betel quid and to their social needs within a specific cultural context. The quitters even re-developed their addiction and returned to the pre-complementation stage.

In summary, besides addiction and withdrawal symptoms, social needs, culture, and psychoactive efficacy lead to continued use by Taiwanese betel quid chewers. Besides the psychosocial and cultural barriers to quitting, the adverse dental effects caused by long-term chewing would hinder chewers during cessation. Cessation programs should thus collaborate with dental professionals and be more accessible. Moreover, chewing betel quid frequently causes oral ulceration, indicating a food safety issue.

Betel quid chewing might cause ulceration of the oral mucosa, and most interviewees considered this disease to be caused by white plaster, the major ingredient in betel quid. The raw material of white plaster is lime, and its sources and processing are described in the book written by Wang [10]. The lime is alkaline, and if the alkalinity is too strong, the chewer’s oral cavity will be burnt immediately. The researcher purchased betel quid containing white plaster at betel quid booths in Kaohsiung and Pingtung County, and they were tested with a pH meter after grinding. According to the results, the pH value ranges from 10.5 to 11, which is similar to the results of the research report by Thomas and MacLennan in 1992 [23]. Similar to previous studies, many interviewees in this study had the habit of smoking or drinking [24,25], and they still smoked and drank when their mouths had ulcers. As a result, harmful chemical substances in cigarettes and wine came into direct contact with injured tissues in the mouth and promoted tissue mutation. If a user continues to chew betel quid with injured oral mucosa, the fibers of the betel quid will rub the injured tissues. As time passes, oral mucosal lesions will naturally result. In fact, a few qualitative studies of betel quid use also state that “betel quid is immersed in hydrogen peroxide” [5,8,10]. The side effect of hydrogen peroxide on the gingiva and oral mucosa has already been verified in the dentistry literature [26]. In dental circles, hydrogen peroxide is used for teeth whitening, and its potential carcinogenicity has been frequently discussed. Generally speaking, exposure of the gingival tissue and oral mucosa to H_2_O_2_ should be avoided. Moreover, it should not be applied to injured tissues or tissues with pathological changes. In addition, in order to prevent possible cancer promoting effects when it is used with the carcinogen DMBA, cigarettes and wine should be avoided when H_2_O_2_ is applied [26].

The influence of betel quid chewing on the oral cavity is a dynamic process. For instance, a betel quid chewing addict might lose a molar due to poor oral hygiene or overuse of the molar. This person might then continue to chew with the other teeth, but as chewing betel quid requires a huge biting force, obvious abrasion will be caused to the other teeth. Moreover, the teeth will become loose and extensive periodontal disease will result. Owing to repeated injuries of the oral mucosa, lesions can be easily triggered. Previous epidemiologic studies often made individual analyses of different oral diseases from a quantitative perspective, which might present a fragmented view that makes it difficult to understand them fully in a dynamic way. In this study, some interviewees suffered from oral cancer several years after quitting betel quid, indicating that long-term betel quid chewing might cause irreversible changes to the tissue of the oral mucosa. Besides providing betel quid quitting courses to help the population, we might institute more critical preventive intervention, such as food safety control and supervision. We can not only guarantee equity among the population, but also clarify whether there were unmanageable confounding factors in previous epidemiologic studies of oral cancer. Furthermore, we suggest that prospective longitudinal studies should examine the relevance of these various factors.

The study has some limitations. First, the sample only included male oral cancer patients of Han ethnicity, and hence is less representative of community chewers and aborigines. Second, interviews conducted in a hospital may have triggered social desirability. Third, some interviewees in this study had declined in speech function due to facial surgery, and their ability to speak was limited. Fourth, only four of the interviewees in this study were at the action stage. As it had been more than ten years since the other interviewees had quit betel quid, this might have caused recall bias. Finally, Taiwanese betel quid is different from that in other countries; a lower addictive level was observed than among Guamanian chewers [15]; therefore, the results cannot be generalized beyond Taiwanese chewers. More qualitative research to explore this addiction globally is needed.

## Supporting information

S1 Appendix Quotes indicating interviewees’ characteristics at various stages. (PDF)
