# Supplementary material for: Qualitative study for betel quid cessation among oral cancer patients
Source: PLoS One. 2018 Jul 17;13(7):e0199503. doi: 10.1371/journal.pone.0199503 (PMC6049897; doi:10.1371/journal.pone.0199503)
Supplement: S1 Appendix — (PDF) [file pone.0199503.s001.pdf]

S1 Appendix Quotes indicating interviewees' characteristics at various stages

Stages	Descriptions
**3.1 Pre-contemplation**	(Note: While "eat betel quid" and "chew betel quid" are both common in daily conversation, chewers do not swallow it, they spit it out after several minutes, just like gum.)
3.1.1 Positive attitude toward psychoactive effects	**M1:** "The feeling of chewing betel quid is that of chewing continuously. When there is something to chew, you are less likely to get bored, you also feel less thirsty, and your mouth is less dry. Chewing then continues." **M1:** "When driving, chewing will reduce boredom and sleepiness, and refresh the mind. Coming to Kaohsiung sometimes, I often felt sleepy if I had no betel quid when driving a car, but I could arrive in Chiayi soon when chewing betel quid." **M1:** "I never thought of abstaining from betel quid in the past, and quit after suffering from this symptom…"
	**M3:** "The job is boring…" **M3:** "I am used to chewing it and feel strange if I don't eat it, as if something was missing. Without chewing it, I feel a bit feckless and want to have some."
	**M4:** "If I feel bored when standing and get bored when I have nothing to chew in the mouth, I will buy and chew it…In other words, I feel less sleepy and thirsty after chewing it."
	**M5:** "It is a good experience to eat betel quid…It makes people feel good mentally. Feeling feckless, you can chew betel quid, and then you will get energy again…" **M5:** "I eat many betel quids when driving a truck from Kaohsiung to and from Taipei back to Kaohsiung. Chewing, chewing, chewing…I need to be wide awake…Otherwise, I will be afraid of having an accident…The traffic was not good in the past, and it often took eight or nine hours from here to there…"
	**M6:** "I need it to stay up late [refresh myself]. Eh…Because of work requirements, I chew betel quid all the time till the next day…" **M6:** "After chewing it, I feel like…Eh…It would be better to chew betel quid when I drive a car, and I won't feel sleepy then."

**M7:** "The more you chew, the more you like it. Sometimes, I even sleep with betel quid in my mouth [laughs]."**M7:** Eh…Why do I want to quit? I will eat it for forty or fifty years before quitting it [laugh]."
**M8:** "I always want to eat it…I feel under the weather if I don't eat it…I am always driving a car, and chewing it gives me high spirits when driving." **M8:** "Nine of ten drivers will eat it, as it can refresh them and prevent them from fatigued driving."
**M9:** "I won't feel cold and often sweat." **M9:** "At that time…I smoke, and that is quite a different feeling." **M9:** "I don't know how to describe this…anyway…I will smoke as long as I eat betel quid, and won't drink when chewing betel quid."
**M10:** "There is nothing to speak of. It does not taste good or bad. That's it and it is just a habit."
**M11:** "Exactly…If…Driving is my occupation; if I need to go to Kaohsiung…If I need to go to Kaohsiung from Taoyuan, I will spend about 100 or 200 TWD on betel quid and chew it when driving on the expressway, as I often drive…drive at night." **M11:** "I mean I will eat it when I feel fatigued or stressed out, and often smoke and chew betel quid at the same time…" **M11:** "It often makes me concentrate and keeps me warm when driving, and of course, this is a personal feeling. I feel like…It can refresh me, and then make me focus." **M11:** "I feel betel quid are because of my job as a driver …. My emotions … I am more …. My personality is more rushed, impulsive, and more emotional. I sometimes lose my temper easily. When I lose my temper, I will naturally want to smoke tobacco with betel quid to calm my emotion." **M11:** "From the first time I ate it to quitting…It has been nearly 33 years, and I don't have the…idea of giving it up."
**M12:** "It can refresh me. When I am driving to deliver goods, I need to pay attention with my eyes, and listen to whether cars behind me honk their horns or anything else with my ears…"

	**M13:** "You often feel less thirsty and more refreshed after chewing it."
	**M14:** "Oh … Betel quid chewing helps the body to stay warm and tolerate the cold weather." **M14:** "I will feel in high spirits when chewing it with my mouth."
	**M16:** "I was driving at that time, driving a…truck; I was chewing it all the time and I've already got used to it…Driving is boring…"
	**M17:** "I didn't eat much in the beginning, and I ate it when my elder brother gave it to me. Otherwise, I wouldn't eat it. But betel quid chewing became a habit for me after I ate it for a time…" **M17:** "I will chew it during work and driving." **M17:** "…I often smoke, eat betel quid and smoke again, which makes smoking more interesting…It seems very pleasant for me to chew betel quid and have a cigarette…It is difficult to describe that…that feeling."
	**M18:** "I will eat some when feeling bored while driving, and often chew it when driving a car…or driving at night. I chew betel quid all day while driving."
	**M19:** "I will buy betel quid and take it while working. I often chew when thinking of it, and otherwise, I will feel sleepy." **M19:** "The more bored you are, the more betel quids you will eat. People fond of betel quids are used to chewing them one after another."
	**M20:** "Riding a motorcycle is boring, and chewing is a habit, not refreshing. It is just like snacks. When chewing, you will feel less bored or sleepy during work."
	**M21:** "It tastes like…It is refreshing. Especially in winter, it will make you warm and less cold. Sometimes, you even sweat…" **M21:** "All I know…It is used to refresh, and that stuff is really refreshing, yes, really refreshing…" **M21:** "I need to work overtime sometimes. When feeling under the weather, I will do just like this, just like what we see now. I either eat betel quid or smoke, and I must find something refreshing."

**M23:** "Because I drive to deliver goods, and became addicted to betel quid when I ate it after my friend gave it to me for the first time. Then I often present it to others, and eat more and more betel quids. I cannot abstain from it." **M23:** "The more I eat, the more spirited I will be…It can refresh me and make me less sleepy…" **M23:** "It is interesting, as if I am chewing something in the mouth. It is really interesting." **M23:** "I have never thought about it …. Just smoking tobacco along with chewing. Chewing betel quids with smoking is more interesting, so I do not want to make any changes … If I did not have this disease [oral cancer], this would not have changed."
**M24:** "Well, I don't know how to describe that feeling! The blending of betel quid and smoking tastes sweet…It is very sweet. I feel comfortable sometimes, and I don't know how to express…" **M24:** "For example, going to work in the north or south, I will think of chewing it when feeling bored while driving, and I often chew it all the way." **M24:** "It is refreshing. Sometimes, when you feel pressured or work against the clock, betel quid chewing can refresh you and relieve your work. It will relieve your work pressure."
**M25:** "It is cold at the construction site…I often feel warm."
**M26:** "I need to refresh myself."
**M27:** "As for its superiority, it makes me spirited immediately…When feeling sleepy sometimes, I will eat one, and that's it." **M27:** "It is a habit…After getting addicted to betel quid, I often chew it." **M27:** "I didn't give it up until I had no teeth, and I quit it because I have no teeth. Otherwise, I will eat it as usual."
**M28:** "I have eaten more and more since graduation…I will feel sleepy if I have nothing to chew in the mouth after I have been sitting in the driver's seat."
**M29:** "I will eat betel quid when bored while driving…I often chew betel quids one after another when

	I feel bored." **M29:** "I never stop chewing it during work till there is none left."
	**M30:** "I will eat betel quid as long as I have time. Just like those addicted to betel quid, I'll chew betel quids once I have some, and eat them one after another in most cases."
3.1.2 Positive attitude toward social effects	**M2:** "Eh…When staying together with friends, I often spend 100 TWD on betel quids and share them with others. We often eat together."
	**M3:** "During work, some elders are all there chewing betel quids…In the beginning, I'm not addicted, but we continued to chew and chew."
	**M10:** "It is like…like…a temple fair, and we eat many at the temple fair…Hey, we have activities." **M10:** "We are…committee members in the temple, and the temple only has 15 committee members. Activities will often be held, and if…When visiting the village, we will certainly prepare things like betel quid and prepare many of them for people like the bearers. We must prepare many for them; otherwise, they don't have the energy to carry the sedan chair."
	**M12:** "I didn't begin chewing betel quid until I did business…yes, did business…I have to engage in social activities. I have to support my wife and children, so I need to engage in social activities."
	**M15:** "All of your friends are chewing betel quid. When you stay together with them, it's not easy to quit."
	**M17:** "When working or discussing business with others, I will eat betel quid."
	**M22:** "I deal with cement, and we often treat each other at the construction site." **M22:** "Ah no … because I have changed my job to air-conditioning, haven't I? In air-conditioning, my seniors chew more betel quid than I do. When both of us have a day out, we cannot do without betel quid … If I go to your home today to install air-conditioning, and you give me some betel quid, tell me how I can change this? It is also a kind of … how should I say this? When you visit a client's house and they are

	chewing betel quid … this is considered socializing ….”
	**M24:** “Because when you are at work, you will definitely buy some for your masters. The so-called master is the person who teaches you. You buy some betel quid, and the master buys some; you need to buy some too. This counts as showing him respect, so you should treat him.” **M24:** “This is the culture of Taiwan, and we have to do three things after getting off work [cigarettes, wine and betel quid] … For instance, at the construction site, they often buy beer during work, and chew betel quid, smoke cigarettes, and drink wine at the same time…”
	**M25:** “We need to establish emotional connections with workers, which is common at the construction site…communication…it is a kind of construction site culture.”
	**M26:** “[Serving in the army] We were all comrades and very interested in betel quid chewing. We were pure at that time, and felt adult when chewing it…We considered it a unique personality.”
**3.2 Contemplation**	
3.2.1 The adverse effects of chewing betel quid	**M2:** “I didn’t think that it had influences at that time…” **M2:** “Eh…My family members told me not to…not to eat betel quid while the children are growing up gradually…Don’t let the children get used to it…”
	**M3:** “I will have loose bowels…betel quid is cold [traditional Chinese medicine considers betel quid to have a cold nature]. (Interview: What influences are there on your work or family?) None. (Interview: No?) Well.”
	**M4:** “Chewing betel quid doesn’t make any difference [no influence on work and family]…One thing is that…If chewing for too long, you will be unable to bite some things. Hey, you can’t bite with your teeth⋯you have to take a rest.” **M4:** “Ah, chewing betel quid sometimes makes holes in my mouth. It is painful. If it is painful, then I quit. If I quit chewing and recover, and see others chewing betel quid, I

will go buy some again. Just like this."
**M5:** "Except the holes in my mouth, it has no other effect." **M5:** "What influence could betel quid chewing produce on the family? The only thing is that my mouth looks ugly…" **M5:** "Ah, others will laugh at me…They often say that my mouth looks black."
**M6:** "The only influence is that I can't open my mouth [laughs]…Ah, I would still chew betel quid regardless, and it didn't feel too bad if I kept chewing."
**M9:** "It looks red. Ah, it has…the flavor of betel quid. At that time, the betel pepper smells fragrant. Finally, my mouth turned out to have fibrosis." **M9:** "It…will cause much harm to the structure of mouth…" **M9:** "Even my teeth are broken." **M9:** "Ah, when chewing relatively 'thick' ash sometimes, I will feel great pain in the mouth…I would be too uncomfortable to eat betel quid that day, because it is…very strange. When chewing something bad, we feel like that…our skin…our tongue is 'penetrated,' you know? It will bite you, and you will find it too painful to eat more." **M9:** "I came across this in my social life … I also think … it is annoying. You asked if I wanted to quit; yes, sometimes. I stop chewing if there are holes in my mouth. When it recovers, I start chewing again."
**M13:** "Sometimes, I will spend thousands of TWD…" **M13:** "My teeth were even broken in the end, and the front teeth were broken." **M13:** "My wife often blames me, but I drive outside…I often feel feckless when not chewing betel quid…"
**M14:** "As for this question…I will stop chewing when I have a toothache. Five or six years ago, when I suffered from a toothache after chewing betel quid, I…Ah, I thought of quitting. So, I quit."
**M15:** "Owing to such red plaster, you will have loose bowels if your stomach is bad. Your excrement can't be hard, and most people will have loose bowels…Unless you have good health, most people will suffer from diarrhea. The red plaster in it is sweet, and we don't know what it's made of. Generally

	speaking, you will suffer from diarrhea, but it also depends. Some persons even go to the toilet six or seven times a day, and that's real…Betel quid with leaves are not as harmful as red plaster." **M15:** "You must suffer from periodontal disease if your teeth are not good, and ulceration is a light symptom. I can't open my mouth now…"
	**M17:** "In my view, I hate betel quid with leaves, because its destructive power is strong…The…the white plaster in betel leaves is…is like lime, which will destroy mucous membrane…." **M17:** "…Sometimes, I feel like I am being watched with strange eyes, as if…as if people thought that you had no…you had no class. I will have such feelings…"
	**M18:** "I often spend much money, which can promote the economy, ha-ha." **M18:** "Sometimes when I think, 'Ah, it is becoming more expensive', I want to quit. Spending a few hundred a day." **M18:** "There are holes in the mouth, and they won't heal."
	**M19:** "I often have loose bowels after swallowing it, since betel quid is cold. Maybe my stomach and intestines are more vulnerable than others'. I often suffer from diarrhea if I drink tea after chewing betel quid." **M19:** "It's your teeth that will be influenced first! Your mouth cavity! Your mouth cavity can't be clean, and then it is the oral mucosa. If you continue chewing betel quid after your mouth is broken, then bad things will grow. People who go to the Stomatology Department after chewing betel quid often continue betel quid chewing after their mucous membrane is destroyed. Then disease attacks us. Otherwise, we would not have cancer cells." **M19:** "Not hygienic! My teeth became ugly and very black, and all of them fell out. The molars were not working, falling out, and were weak … Betel quid chewers have a weak lower jaw. It is difficult to chew a piece of meat even you want to. I can chew betel quid no matter how hard it is, but I cannot chew meat …."
	**M21:** "Yes, I have [the idea of quitting], but I think that I don't have a strong addiction and that my life

	is normal. Moreover, I also do mouth care, so I don't have a strong desire to quit it…"
	**M24:** "The older generation says that you should not quit after getting addicted to betel quid. Otherwise, your teeth will be softened…So I decide to quit gradually." **M24:** "After chewing betel quid for a long time, my teeth become loose, and my gingiva swells up…If you continue to eat betel quid after your mouth swells up, you might suffer from great pain…Ah, my lips on this side are ground by betel quid, and betel quid chewing has led to fibrosis…"**M24:** "Every time your mouth is broken, you can't have meals as usual, so I quit."
	**M25:** "It looks ugly and my body becomes bad…My teeth are black." **M25:** "When meeting others for work, the impression is not very nice. We are considered the middle level at the construction site, but there are more senior levels. When talking to people, or whatever it is, the impression is not very good."
	**M28:** "Your mouth looks dirty…Its appearance is bad. Sometimes, I think it quite impolite to talk with my customers with such a mouth." **M28:** "Sometimes, my wife also chatters that my mouth is so ugly."
	**M29:** "I haven't quit after trying dozens of times." **M29:** "It makes people suffer from diarrhea…Everybody is like this, and often goes to the toilet. The more you eat, the more frequently you go to the toilet." **M29:** "When I eat the most, I will spend one thousand a day…"**M29:** "It costs a lot, and often damages the mouth. I will quit when my mouth is broken and cannot eat."
	**M30:** "It will damage the teeth and gingiva, and then lead to oral lesions. Besides, it is no good for the health, and you may suffer from diarrhea sometimes…These are common problems." **M30:** "…I always think of quitting betel quid, but I can't make it…"
	**M10:** "It is not delicious, but popular. Ah, it is popular, and I eat when others chew…" **M10:** "It doesn't have any influence…There is no influence, but…after hitting the jackpot [suffering from oral

	cancer], I think that…I should not eat anymore, so I quit! Completely quit! Completely quit! Even quit cigarettes and wine! **(Discrepant case ➔ no bad influence)**
	**M20:** "After biting betel quid for a long time, when you encounter something hard sometimes, your enamel is not hard enough to chew it…Being under abrasion for a long time, it must be vulnerable, and tooth lesions will occur easily. Your mouth is comparatively vulnerable, as with gingival inflammation." **(Negative case ➔ no intention)**
	**M22:** "Ah, maybe my stomach is not good. Ah, sometimes, you will swallow the betel quid residues, just like the balloons. Sometimes, you will swallow it if you are careless…Residues will be swallowed more or less, and it is bad for your stomach…"**(Negative case ➔ no intention)**
	**M23:** "The mucous membranes of my mouth are very stiff, but I have to eat…The mucous membranes in my cheek are quite thick, and it feels strange to eat something…The uncomfortable thing is that too many betel quid residues stay in my stomach, and I have to wash my stomach and remove the betel quid residues…" **(Negative case ➔ no intention)**
3.2.2 Addiction	**M5:** "I ate betel quid when I was a juvenile, but I was not addicted. I felt well without betel quid at that time. I would eat it when I had betel quid, and it did not matter to go without betel quid. Ah, I chew it every day when driving a tractor…"
	**M8:** "I kept chewing unless I was asleep at that time, and it seemed that I stopped chewing only when chewing meals and sleeping. I always chewed betel quid the rest of the time." **M8:** "Medical reports often say that we should take less cigarettes, betel quid, and wine. Otherwise, I would not have thought of quitting. Especially betel quid; I feel strange if I don't eat betel quid when others do." **M8:** "Ah, I began to chew betel quid because I thought it was interesting to chew betel quid when I saw others doing it. After several times, I wanted to quit, but…I couldn't…"

**M12:** "It smells bad, and is not tasty [laughs]. I felt like throwing up after chewing it, and learned to eat betel quid the first time… (Interview: What did you feel later?) It does not…does not smell so bad, but…almost the same…it's always that taste after I tried ten or twenty times. It has a bad smell…but later I got addicted, and cannot quit." **M12:** "Well…I was addicted to it at that time, and many people told me to quit, but I couldn't."
**M13:** "Ah, it is a habit, and I feel strange without chewing it. In a general sense, I am addicted to it." **M13:** "…I eat more and more." **M13:** "I keep chewing except when I sleep…"
**M15:** "I felt dizzy and my heart beat faster when I ate betel quid for the first time, and also the second time and third time…I got addicted after chewing it three or four times, and often think of it when I don't chew."
**M18:** "A habit, if it is a habit, you will not know what its pros and cons are anymore. I do not know, but it is a habit. I feel like having something to chew when driving at work, chewing one after another. I do not feel anything much, but I just like chewing. When my mouth is empty, I feel like finding something to chew."
**M19:** "Betel quid was cheap in the past, and I got addicted when I treated it as a snack. Addiction is a habit, and it is hard to quit, just like smoking, which is also difficult to quit. It is hard to quit both chewing betel quid and smoking cigarettes." **M19:** "I chew betel quid all day except during sleep, and chew every moment… Sometimes, I even sleep with betel quid in my mouth, because it has become a habit."
**M26:** "Everyone including my wife opposed it strongly, so we had many conflicts about it…But I chew it for life's pressures. I don't have other hobbies. After chewing betel quid, I don't notice the working hours so much, and the work does not seem very long…" **M26:** "I wanted to take a rest after getting off

	work, so I would stop chewing betel quid. I chew betel quid after work and even while sleeping at the end. In the end, I'm afraid of others knowing my mouth, so I narcotize it with betel quid. Otherwise, it will ache. The mucous membrane of my mouth has been broken. I don't eat during sleep, but when I begin to chew it after getting up in the morning, I often feel great pain. It will grind the mucous membrane of mouth, which hurts. But later, I won't feel any pain the whole day…So I continue chewing it." **M26:** "In the end, I live by holding betel quid in my mouth."
	**M29:** "No. I'm just addicted, and it has no relation to that [improvement of work efficiency]."
	**M30:** "In the beginning…I didn't eat much in the beginning, but I chewed more and more after getting addicted to it." **M30:** "I often chew betel quids one after another, just like smoking cigarettes one after another. After getting addicted, I would eat betel quids one after another." **M30:** "That is…I eat more and more as time passes, and eat more and more when staying up late." **M30:** "You will eat betel quids after waking up, unless there are no betel quids when you wake up. Then you will go out to buy betel quids after having your meal. Just like this." **M30:** "I often held betel quid in my mouth in my sleep during the severe period. Later, I changed this habit." **M30:** "Sometimes I want to, but cannot quit. Anyway, many people, like your mother, have been telling you, not to chew betel quid, but you still cannot quit. Since betel quid chewing has already become my habit, I have to chew them." **M30:** "I think of quitting betel quid every day…It would be better not to eat betel quid, and I tell myself not to eat betel quid every day, but…but I still cannot quit. I still buy betel quid." **M30:** "This … regarding betel quid chewing … it only has a hundred harms, and no benefit. Even if you wanted to, you cannot give it up, even if you wanted to stop chewing every day. You want to stop chewing tomorrow, but … but you will just continue chewing. If you think about not chewing tomorrow, before bed you think about not chewing any betel quid tomorrow. You will still … you will still chew some the next

	morning.”
	**M7:** “When waking up and going to work after waking up…anyway, when my eyes are open, I will eat betel quid and brush my teeth with betel quid. In other words, I use betel quid to brush my teeth…Betel quid is used as a toothbrush. Before I go to bed, I also put betel quid into my mouth.” **(Negative case ➔ no intention)**
	**M27:** “I keep chewing either during work or not…Sometimes, I sleep with betel quid in my mouth.” **(Negative case ➔ no intention)**
3.2.3 Physical problems	**M3:** “I suffered from *Vibrio vulnificus* [in my mouth] one time, and recovered after the pus was taken out.”
	**M12:** “I have had a stroke …I have been quitting for around three to four years….”
	**M17:** “I have to collect my medication from the Hepatology Department, and my fatty liver index is relatively high. Since it became relatively high, my doctor has told me not to chew it anymore and also to quit tobacco ….”
**3.3 Preparation**	**M2:** “You mean…Afterwards? Afterwards, I got ill, oral cancer…”
	**M3:** “I felt strange in my mouth, so I quit [stopped chewing it].”
	**M4:** “I began to chew gum.”
	**M5:** “At the early time, Frank Hsieh of Kaohsiung City was in his second term…He ran for the second term…On the night when he was elected, I quit. I was the deputy director-general of his support group, and people laughed at me [my black mouth]. Ah, I made a vow, ‘If Frank Hsieh were selected, I would…quit.’ On that night, I quit.”
	**M6:** “I couldn’t open my mouth [OSF]…” **M6:** “Ah, I stopped chewing, and quit…”
	**M9:** “Sometimes, I wanted to eat less…Working in that environment, you can’t quit it.” **M9:** “I will

gradually chew less and less.”
**M10:** “I hit the jackpot [oral cancer], so I have to quit…” **M10:** “just stopping chewing it.”
**M11:** “…After suffering from oral cancer…Ah, I really quit it and completely quit…I got divorced from…was separated from it.”
**M12:** “I completely stopped. I won’t eat it again.”
**M13:** “I received chemotherapy…my daughter didn’t allow me to eat it again…”
**M14:** “Once I stopped, I never thought of chewing it again.”
**M17:** “Chew gum…”
**M18:** “I stopped chewing it, just so.”
**M21:** “You will not believe it, even if I say it. I was thinking when driving, I was thinking about how much I had spent on chewing betel quid in these many years, and on smoking tobacco. Why I do eat betel quid? … I then threw them away and quit chewing them from then on. If people force you, you will experience a rebound. You will follow this if it comes from your inner self ….” “You often feel bored when driving on the expressway, and then I thought about why I ate betel quid and why I smoked. (Interview: Throw it) I threw it outside the window, and stopped chewing that! Many people don’t believe, but I really quit like this.”
**M22:** “My elder bother found that a tumor was growing in me, and it was stiff…I visited Doctor Guo in the otolaryngology department, and he suggested an operation…”
**M23:** “Owing to the esophageal cancer…The doctor told me not to eat betel quid, smoke, or drink wine. Ah, I take all three…”
**M24:** “When my teeth were loose but had not fallen out, I quit.”
**M25:** “In fact, I didn’t have some special ways. Just stopped chewing it.”

	**M26:** "A sarcoma grew in the mucous membrane of mouth…My teeth can touch the sarcoma, but it doesn't ache. I knew it was bad, so I quit."
	**M28:** "Stop chewing it if you don't want it."
	**M29:** "Stop chewing it, and there is no special way."
**3.4 Action**	
3.4.1 Successfully quit betel quid	**M2:** "[Laugh] Just restrain yourself. (Interview: Do you have other methods?) No, no…"
	**M4:** "I didn't quit it until…last year when I visited a doctor for oral cancer. I quit when I visited Doctor Wu."
	**M5:** "Ah, it doesn't make a big influence when you stop chewing it…It is all about willpower…"
	**M6:** "Ah, it is the same as quitting smoking…It is all about willpower. Stop chewing it when you make the determination to…No, I don't chew gum…"
	**M8:** "Ah, I quit betel quid, smoking, and wine when suffering from oral cancer. Ah, the doctor also said, 'Quit them if you hope to live.' So I quit them all. I felt painful at that time, and wanted to eat betel quid and smoke cigarettes within five days. Ah, I…I think…According to the doctor's suggestion, they are bad things. Ah, I quit them all finally."
	**M10:** "I was afraid to eat it when I hit the jackpot…I was a little bit addicted…But it was not severe…I can still survive without it…Some people cannot survive without it…"
	**M11:** "Use willpower, just like smoking. Even until now, I still want to smoke tobacco. When I smell tobacco, I still have that urge to smoke. How amazing it would be to have just one smoke. However, I used my willpower to overcome my addiction to smoking. Similarly, for betel quid, I used willpower to quit and it worked. When I said no, it meant no." "A friend of mine in the in-patient ward couldn't stand it, and he also ate betel quid. Doctor Wu said, 'Ah, how dare you eat betel quid?' Ah, I also

scolded him, and said, 'Mr. Mo, are you still chewing betel quid?' Alas! His willpower is weak, so he began to eat betel quid again and came back to the hospital. So I dare not eat it, and I am afraid." **M11:** "When reflecting on something, I will buy gum, as if it was like…(Interview: To replace betel quid…) Well, Yes, it's true. I personally think that it can make me concentrate…When driving a car, I will also chew gum. Because I drive, when chewing something…eh…I can concentrate…" **M11:** "I don't eat gum frequently. Sometimes…I didn't use gum to quit betel quid. I was afraid when receiving an operation, since an operation is a *very* difficult thing. Especially in the intensive care unit, I felt extremely tired. So health is…Ah, I don't want to go back to the intensive care unit again, so I quit it."
**M12:** "…I ate more and more when I tried to quit. (Interview: The last time you quit…) I'm determined to…I'm determined to quit it. I have the perseverance…No, that is not good." **M12:** "It needs determination. Some friends may deliberately make fun of you and give you some. I will say no, and it means no. Because… doing business until having a stroke, I told myself not to chew any betel quid. (Interviewer: How about when people pass them to you?) When I said no, it means no. Because I would tell him I had a stroke."
**M13:** "My daughter would scold me. She asked me why I ate it again and whether I wanted to visit a doctor." **M13:** "I felt strange in my mouth if I did not eat betel quid, and wanted to chew betel quid all the time. Later, I tried to control myself, and got used to it in more than one month." **M13:** "I'm not used to chewing [gum] all day…Later, I quit."
**M16:** "I didn't quit until I visited here. At that time, I suffered from leukoplakia, so I had to quit…So I directly quit…I'm determined to stop chewing it again, and that's it…"
**M17:** "I never ate betel quid again after I quit, and I quit once and for all…" **M17:** "…I'm not addicted." **M17:** "Ah, they wanted to treat me, but I refused and said that I wouldn't eat it anymore.

They wouldn't force me to eat, because it makes no sense…"
**M18:** "I don't receive any examinations, and I just know that there are holes in the mouth…" **M18:** "In the beginning, I will chew gum, but later I stopped and got used to it." **M18:** "I quit when I told myself to, and there is nothing that cannot be overcome. The most important thing is that you want to quit in your heart, and you can't quit it if others tell you to…"
**M19:** "Oh, my method does not work on many other types, only this. Whenever you want to chew betel quid, quickly brush your teeth and make them feel clean. With a clean mouth, you are less likely to eat anything. Even if people ask me to eat peanuts and chew tea leaves, it will not work. I have tried quitting several times by those methods, which were not effective." **M19:** "I didn't decrease the amount! You can't quit by decreasing the amount! You have to quit it directly." **M19:** "I quit because I had no money to buy it, and then I succeeded. Hm, I had no job and money, so I must quit betel quid."
**M21：**"I'm a little bit addicted, but it is not severe…it is tiny, and my addiction is tiny. I want to chew betel quid, but the quantity … I will control the amount and I can refrain from it. I can survive without it." **M21:** "I completely stopped chewing betel quid. Ah, I ate gum for several days, and quit maybe because I thought it did not taste good…" **M21:** "It should be said the social life and work load have been reduced. When you were young, like at 20 and 30 years old, you might have had a large workload or needed to earn money; you needed to energize yourself. Turning 40, when turning almost 40 years old, your income and everything will stabilize and you not need to put in that much effort. Of course, if you do not have much physical output, you will not need to energize yourself to an excited state …." "I should say that most people chewing betel quid engage in labor work. Because they have to make a living and earn money, they need to work overtime and have to keep their spirits up…That is a kind of…a kind of sadness…That is a kind of sadness. If everybody's environment is good, no one would

like to get refreshed with betel quid…We can't do anything, and that's it…"
**M22:** "[After receiving an operation] Ah, I directly quit, and even my friends cannot make me eat it again…"
**M25:** "I had a stable job and stable customers at that time. I just needed to make an inspection tour at the construction site. If [the workers] need some, I will prepare some for them. I don't eat anymore." **M25:** "I seldom eat betel quid, and dropped off gradually."
**M26:** "Actually, I'd already known this two months before the examination. I stopped eating it in March 2014, and I knew that my mouth could not be saved. I know this fairly well, since I had chewed so many betel quids. It must be oral cancer, so I have to face it by myself…"
**M3:** "Ah, I thought I had to quit at that time, and felt…My mouth was broken more and more frequently. Ah, I quit then." **M3:** "Eh…I felt that my mouth…my mouth was often broken, broken, recovering, broken and recovering. When I recovered in one or two days, I quit. I felt my tongue burning the second month after quitting it…Finally, I took some anti-inflammatory drugs. One week later, it didn't take effect. Finally, I felt a stiff place. When I visited the clinic, they asked me to undergo a pathological examination. The examination results indicate tongue cancer." **M3:** "Eh…with such symptoms…I must quit." **(Discrepant case ➔ cold turkey, not entering the maintenance stage)**
**M20:** "It is because you cannot buy them overseas, which has happened twice. You need to do business overseas and cannot buy any, so you will quit it naturally when you return." "I could not buy any when going abroad, two times." **M20:** "I suffered from oral cancer…I quit after learning the result, and quit naturally this time. You can't ignore your own health, right?" **(Discrepant case ➔ cold turkey, not entering the maintenance stage)**
**M28:** "I just quit. To tell the truth, it is not easy to completely quit it. When my colleagues bring me

	some, I will eat one or two.” **M28:** “With good friends or customers, sometimes customers who are very important, I cannot decline…” **M28:** “Sometimes, one pack might include 10 or 12 betel quids, and I will eat three or two on the day I buy the pack.” **M28:** “I don’t feel very nervous but sleepy…not concentrated.” **M28:** “I will stand up and take a walk or ask for one from others. I just like the feeling of betel quid in my mouth…I will put it into my mouth and chew just once or twice. In the past, my mouth often became red after minutes of chewing betel quid. Sometimes, I will spit it out after chewing for a short period.” **(Discrepant case ➔ decreasing the amount)**
	**M30:** “I eat betel quid…I don’t have a last time. I didn’t eat it when I was hospitalized, and still chew it now…I could not quit immediately, and my teeth would fall out and swell up.” **M30:** “I didn’t eat it when I was hospitalized. When I was hospitalized for the first time, I stopped chewing betel quid for more than one month, and did not eat it after I left hospital…My second hospitalization lasted for…more than ten days, seventeen or eighteen days. Anyway, I didn’t eat it when I was hospitalized. After leaving the hospital, I quit for a certain period.” **M30:** “I have oral cancer on the right, and can chew betel quid on the left side. Holding such an idea, I will continue chewing it.” **M30:** “I want to quit, but I can’t…Sometimes, I still want to have some betel quids. That’s it…” **(Discrepant case ➔ decreasing the amount)**
3.4.2 Loss of oral functions	**M4:** “Ah, I fell ill, had no teeth to chew it, and all of my teeth dropped out.”
	**M7:** “I can’t eat it anymore; I suffered from oral cancer and my teeth fell out. I have to quit.”
	**M9:** “My tongue will hurt [tongue cancer], and I can feel pain when chewing betel quid. Ah, if chewing something else after chewing the betel quid, I will have a different feeling. I often feel pain when chewing a meal or hot soup. Ah, a certain period later after I quit, it wouldn’t hurt.”
	**M10:** “My teeth are bad, and you have to pull out all of them before the examination. After you pull out

the teeth, you can't eat it anymore."
**M14:** "Just oral pain. The teeth were weak when chewing and I was unable to continue to chew, and that made me quit."
**M15:** "…I couldn't open my mouth before…it was not as severe as now. Almost 11 or 12 years later, my mouth could not open, and I knew something was wrong…After visiting the doctor, I had to receive [an operation]…I didn't want to, so I decided to delay." "In the end, I was … my mouth could not be opened, and that made it difficult to squeeze any betel quid in, you know, then I chewed less. I tried to squeeze them in, but had less than 10 a day. It hurt when chewing …." "At that time, you will feel great pain when chewing…I seldom ate betel quid at that time; because you are unable to chew, you will stop thinking of chewing."
**M23:** "Your mouth cannot chew after having esophageal cancer and the feeding tube will be inserted here for feeding; how can you chew betel quid?"…"I can't eat and my mouth can't bite, so I have to be force-fed…Now, I'm going to receive chemotherapy, and only have four teeth. So I can't chew and dare not chew now…"
**M24:** "Sometimes, if you have a toothache or your mouth is broken, you have to stop betel quid chewing for three or four days before you recover, as your gingiva will swell up. Ah, you have to take a rest…" **M24:** "I started chewing on this side. When the teeth had fallen out and I could not chew, then I moved to the other side. When that side also started loosening up, and I was also in pain, I decided to stop chewing …." **M24:** "The teeth are loose, and I'm afraid that my teeth will fall out one day, so I hope to quit. I am galled every time I eat it and the side in my cheek has been…the mucous membrane inside has been gone, so my mouth will be broken every time I eat it. So I decided to quit." **M24:** "Every time you want to put one into your mouth, your mouth will get broken. Of course, you have to

	quit.”
	**M27:** “I didn’t quit until all of my teeth fell out, and I can’t chew it without teeth.”
	**M29:** “I didn’t quit until all of my teeth were pulled out [oral cancer]. I cannot chew anymore.”
3.4.3 Relapse	**M3:** “My job is boring and all of my friends are chewing it. Seeing others chewing betel quid, I often become greedy in my mouth, so I continue.”
	**M4:** “Seeing others chewing betel quid, I often become greedy in my mouth.” **M4:** “Generally speaking, I can keep not chewing it for half a month.”
	**M5:** “(Interview: Do you have any other way?) No, I just quit and stopped chewing it. Ah, I will think of it…but…it’s all about willpower…You have to quit it. When others bring me some, I chew it again.” **M5:** “Err …Sometimes several months, sometimes I relapsed after several weeks. This is just like taking medication. You will think about it when you stop having it. The willpower helps to stop you from chewing … Ah … It is very boring every day. If for others … my friends were talking and chewing, and offering some betel quid. I was embarrassed to turn him down...”
	**M9:** “My friend will treat me, and we often hold each other accountable. He would say, ‘Ah, you are chewing betel quid, but you said you would quit…’ And I would take some more and put it into my mouth.”
	**M10:** “Others are chewing, and I often feel embarrassed if I don’t eat, right? Anyway…I will chew, just as before…When we make friends with each other, all of them are chewing betel quid, and if I don’t eat, I will…become a person not of the same clan. Then I became a person not of the same clan, and they would say, ‘You don’t eat betel quid, so you shouldn’t be together with us.’ That’s it…”
	**M12:** “I have tried quitting betel quid chewing dozens of times. However, chewing increases every time after quitting. I ended up having more every time after I quit, and this cycle goes on. If I quit now,

	I will still want to chew, and will even have a lot more than I did before … The more I quit, the more betel quid I will chew."
	**M13:** "I tried to quit before, as my wife told me to. Ah, I couldn't quit it, and began to chew again a less than a week later." **M13:** "This means that it is like drinking alcohol, people have said … people in the past have said that it is same as alcohol poisoning."
	**M18:** "It's about one month, and I began to chew again." **M18:** "I will feel strange without chewing it. Moreover, all of my friends are chewing betel quid. In other words, all the people in my working environment are chewing betel quid." **M18:** "Sometimes, I will take some to others, and sometimes, others will bring me some."
	**M19:** "I often become greedy in my mouth. Seeing others chewing, I can't control myself…It is mainly a habit, and it's hard to quit."
	**M25:** "Sometimes, it's hard to refuse when others give you some at the construction site."
	**M28:** "Have tried to quit too many times. The craving comes after quitting almost three days to one week. I am working at this construction site where every colleague chews them. We treat each other, so I feel embarrassed if I do not take it. So I will take it and squeeze them into my mouth." **M28:** "We've taken such a job. We do hard work and all workers are chewing betel quid…"
	**M29:** "Seeing a betel quid booth outside…I couldn't help buying some…I just want to eat it." **M29:** "Not able to quit. My mouth becomes dry. The tooth will become swollen, and that hurts. The pain is relieved when chewing …." "It hurts if I stop chewing, but I won't feel any pain when I continue chewing…Chewing slowly, I won't feel any pain in the end."
	**M30:** "There is no way to quit. This is me … I have no idea how to quit. If you asked me to try chewing gum, I would not get used to chewing gum, I would also not get used to chewing candies.

	Also, you can buy something … I forget … lico … lico … licorice, right! Buying licorice cannot help, I cannot get used to it. It is the same with sugar cane … Every day you think about not having any the next day, and that you will not have any tomorrow morning. However, you will still buy betel quid to chew as usual when you wake up. Many people have said not to chew betel quid, but I will not listen. They said their own things, and I continue my chewing.” **M30:** “Seeing others chewing betel quid, you also want to chew some. Anyhow, when others are chewing, you just follow them. On the road, some workers and colleagues do like this, and your colleagues are also chewing betel quid during work.”
**3.5 Maintenance stage**	
3.5.1 No withdrawal symptoms and getting rid of the addiction	**M1:** “Ah, I completely quit and never eat again. The doctor told me not to chew, so I stopped chewing. Ah, I completely stopped.” **M1:** “Ah, I have never eaten any betel quid up to now, and it has been about ten years…”
	**M2:** “Yes, I quit when I received the examination.” **M2:** “I’ve never eaten any betel quid till now, and it has been about two years…”
	**M4:** “As for the last time, I quit when visiting Doctor Wu. I never eat any, and even quit drinking. I quit once.”
	**M6:** “I quit once, or what [laughs]…It’s ten years…”
	**M7:** “I don’t take those things, cigarettes, wine, and betel quid. I quit them completely.” **M7:** “It has been more than two years…”
	**M8:** “It has been about four years since I quit it…”
	**M10:** “… I say no, and that means no. If you want to, you can chew as much as you want. If you rush to have it, then just go! I have no method to persuade you guys.”
	**M11:** “No, no, I don’t feel uncomfortable. As for smoking…I still have an addiction, but I’m not

addicted to betel quid. Betel quid has no side effects, and to stop chewing betel quid won't lead to any bad consequences." **M11:** "Because I … honestly speaking, I was scared … This cancer is really … this … my daily routine had been completely changed. You dare not eat what you like. For myself, I was engaging in my social life, and everything had been changed. Ah … [Sighs]. Therefore … betel quid chewing is really not good. Life and daily routine will have changed …."
**M12:** "No, it is ok when you get used to it. I mean it is ok when you get used to it after quitting [withdrawal symptoms such as absent-mindedness and sleepiness]."
**M13:** "Coming here for chemotherapy… My daughter did not let me chew betel quid… After having a strange feeling for almost one to two months, this feeling went away after getting used to it……Quitting causes a strange and itchy feeling in the mouth; it creates a craving for chewing. Later, I kept tolerating and tolerating this until I got used to it after a month. I may feel [quite tired], and approximately … more than a month, everything went back to normal."
**M14:** "Well, I don't want to chew, and then I won't think of it. When I don't think of it, I won't feel anxious." "In the past, I got used to chewing. I would find it, and we had this thought. Now, I will not think about it as I have stopped chewing; I have no intention to buy it or whatever."
**M15:** "I can't chew…It hurts…"
**M16:** "I won't think of chewing it again after quitting, never…"
**M17:** "As far as I know, some people's gingiva will ache after they stop chewing betel quid. They told me. But…mine didn't. When I stopped chewing betel quid, my gingiva was ok. I never felt uncomfortable."
**M18:** "No [symptoms of quitting]. I was not used to it when driving a car in the beginning…But several years later, I don't want to chew betel quid."

	**M19:** "I just, just want to have a taste. It is nothing, and all people can be greedy in their mouths. I would still dream of chewing betel quid two or three years later after quitting. I don't know why I dreamed about it after quitting."
	**M22:** "It has been several years since I quit, maybe five years. I don't want to eat betel quid, and never think of things like that. Ah, to tell the truth, it is much more difficult to quit smoking…" **M22:** "I won't eat it even when my friends bring me some."
	**M23:** "I often felt strange when I stopped chewing betel quid, in the beginning…as if I hadn't eaten my breakfast, and this was strange…I've never eaten any after quitting it, and I can't eat it, since I have no teeth."
	**M26:** "It has been less than one year since I quit… (Interview: Will you chew it again in the future?) Never! (Interview: If someone brings betel quid to you…) I won't eat it…Now, when I see people chewing betel quid, I often feel pity for them even if they don't have a symptom [oral cancer]. I have a merciful heart, and that's it."
	**M27:** "I felt strange if I didn't eat betel quid…I would become droopy, but as time passed, such feelings faded away." **M27:** "Anyway, I can't eat it without teeth."
	**M29:** "[In the beginning] I always felt thirsty and droopy, which is an idle state…"**M29:** "How can I eat without teeth?"
	**M24:** "I am not addicted." **M24:** "(Interview: Are you nervous and anxious? Do you feel strange? Are you sleepy?) No."
	**M25:** "No [withdrawal symptoms]." **M25:** "In the end, he always brought me some, but I didn't accept it. I never ask for some from others."
3.5.2 Recurrence	**M2:** "I forgot [answering with a smile]. I was feeling annoyed, or whatever it was during that period of

	time … and started chewing again. Chewing and then … it continued … and continued chewing, and it did not stop.”
	**M20:** “Shortly after I went back, my friends would bring me some, knowing that I ate betel quid before. Then I would think of biting one or two…It was ok in the beginning, but as time passed, I got addicted to it again…” **M20:** “As for the second time, it happened one or two weeks later after I went back, less than one month…”
	**M24:** “Sometimes when I work at the work site, people treat you... Chewing again will result in addiction. Just like this ….”
	**M17:** “I don’t eat it now, but…I ate it again after quitting when I came to the south and contacted the aborigines. But…the reason why I bought betel quid is that I needed to treat them. Ah, I also ate sometimes, occasionally. However, it did not last long, just occasionally.” “… because I have a godbrother who is an aborigine. He is the chairman of the village representative council, so like him, he sometimes runs elections or something. I will visit each of their villages, for example ten villages in Sanmin Township. We will even visit mountain villages in Pingtung County. I will chew some during that time. If I do chew, but … that would be … just for a very short period of time.”
